# Nearly Complete Genome Sequences of Two *Mimivirus* Strains Isolated from a Japanese Freshwater Pond and River Mouth

**DOI:** 10.1128/genomeA.01378-16

**Published:** 2016-12-08

**Authors:** Masaharu Takemura, Tatsuya Mikami, Shingo Murono

**Affiliations:** a Laboratory of Biology, Department of Liberal Arts, Faculty of Science, Tokyo University of Science, Shinjuku, Tokyo, Japan; b Graduate School of Mathematics and Science Education, Tokyo University of Science, Shinjuku, Tokyo, Japan

## Abstract

Members of the *Mimiviridae* family are large DNA viruses that infect *Acanthamoeba* cells. Here, we report the genome sequences of two new *Mimiviridae* family members, isolated from water samples from Shirakoma Pond and the mouth of the Arakawa River in Japan, with nearly complete genome sizes of 1,182,849 and 1,182,801 bp, respectively.

## GENOME ANNOUNCEMENT

Large complex DNA viruses of the family *Mimiviridae* have very large icosahedral particles of 750 nm in diameter, including dense surface fibers, and encode the largest genomes of around 1.2 Mb in size (1–3). Here, we report the nearly complete genomes of two isolates of *Mimiviridae*, isolated from the freshwater of Shirakoma Pond, located in Nagano, Japan, and the mouth of the Arakawa River, which is close to Kasai Rinkai Park in Tokyo, Japan. The isolates were named *Mimivirus shirakomae* and *M. kasaii*.

Water samples were collected from the pond and the mouth of the river. After mud was removed by filtration (filter paper 43; Whatman Plc.), samples were further filtered (1.2-µm pore size; Millex-AA, EMD Millipore). Filtered samples were supplemented with 4% rice media (4). After incubation for one month in the dark at room temperature (4), the samples were mixed with a suspension of amoeba cells in peptone-yeast-glucose (PYG) medium and were added to this viral solution, which was divided and cultured on a 12-well culture plate at 26°C. After several days, we found that amoeba cells in several wells had delayed proliferation and were almost round. The supernatant of culture in one of these wells was inoculated to fresh amoeba cells in a 25-cm^2^ culture flask. After two days, rounded amoeba cells were harvested. Then, the supernatant was stored at 4°C as an isolated virus solution. After virus cloning (5), genomic DNA of *M. shirakomae* (1.1 µg) and *M. kasaii* (1.2 µg) was prepared from PYG culture media including viral particles using NucleoSpin Tissue XS (Macherey-Nagel GmbH and Co. KG) according to the manufacturer’s protocol. A DNA library for sequencing was prepared using a TruSeq Nano DNA LT library prep kit (Illumina, Inc.), and sequencing was performed on the HiSeq 2500 platform (Illumina, Inc.). Edena software was used to assemble 58,319,134 reads into nine contigs with an average length of 131,428 nucleotides (nt) for *M. shirakomae*, and 71,243,710 reads into eight contigs with an average length of 147,850 nt for *M. kasaii*. The maximum contig lengths for *M. shirakomae* and *M. kasaii* were 680,266 nt and 908,954 nt, respectively. The total length of the nine contigs in *M. shirakomae* was 1,182,849 nt, and the total length of the eight contigs in *M. kasaii* was 1,182,801 nt. Prediction of the coding regions of these genomes was conducted using Prodigal version 2.6.3, and prediction of tRNAs was conducted using tRNAScan-SE version 1.23, according to the manufacturers’ protocols. The prediction of gene function was conducted using NCBI BLASTp in the NCBI NR and COG databases.

Genome analysis showed that these *Mimivirus* strains have genomes of around 1,182 kb, which are approximately equal to the genome sizes of other *Mimiviridae* lineage A members (2, 6–9). These draft sequences of both isolates are predicted to have 994 coding sequences and four tRNA genes. Genome comparison revealed that *M. shirakomae* and *M. kasaii* most closely resemble *M. bombay* (9) in the genus *Mimiviridae*.

### Accession number(s).

The nearly complete genomic sequences of *M. shirakomae* and *M. kasaii* have been deposited in DDBJ/ENA/GenBank under the accession numbers AP017645 and AP017644, respectively.
